# Efficacy of a Single Dose of Pregabalin on Signs of Anxiety in Cats During Transportation—A Pilot Study

**DOI:** 10.3389/fvets.2021.711816

**Published:** 2021-09-01

**Authors:** Terttu Lamminen, Mira Korpivaara, Minna Suokko, John Aspegrén, Clara Palestrini, Karen Overall

**Affiliations:** ^1^Orion Pharma, R&D, Espoo, Finland; ^2^Department of Veterinary Medicine, University of Milan, Milan, Italy; ^3^Department of Health Management, Atlantic Veterinary College, University of Prince Edward Island, Charlottetown, PE, Canada

**Keywords:** feline, fear, travel, carrier, clinical, pregabalin, anxiety, transport

## Abstract

**Objectives:** The aim of this clinical pilot study was to evaluate the dosage, efficacy, and clinical safety of a single oral dose of pregabalin in cats that experience fear and anxiety when placed into a carrier and transported by car.

**Methods:** Thirteen client-owned cats were enrolled in a blinded, randomized, crossover study with three treatment days approximately 1 week apart. The cats were assigned to receive pregabalin oral solution at dosages of 5 and 10 mg/kg and placebo in a randomized order, one treatment per week. Treatment was administered ~90 min before placing the cat into a carrier and starting transportation. Efficacy was assessed by the owners using a categorical scale and, based on video recordings, by an external observer, both blinded to the treatment.

**Results:** Owners assessed that cats given pregabalin displayed less vocalization, restlessness, and panting during transportation than did cats given placebo. Correlation between owners' and external observer's assessment of the overall treatment effect was good (0.63, *p* < 0.01), which confirms the owners' ability to observe reliably their own cat's behavior. Transient mild ataxia was the most common adverse event reported. The human commercial formulation used in this study was found difficult or very difficult to administer by 79% of the owners.

**Conclusions and Relevance:** Based on results of this pilot study, a single oral dose of pregabalin was well tolerated and decreased signs of anxiety and fear associated with car transportation in cats, as evaluated by blinded owners and external observer. The use of pregabalin prior to traveling may improve cat welfare and compliance for transportation. Further studies are needed to investigate the use of oral pregabalin in cats to alleviate signs of anxiety and fear associated with transportation and sequelae, like veterinary visits, and to develop a more user-friendly formulation.

## Introduction

Cats are common pets, occupying approximately 25% of households in the United States (1) and Europe (2). However, there is concern that cats often lack both adequate preventative and acute veterinary care (3, 4). One of the main reasons for this welfare concern is that cats are often too challenging to transport (3, 4). Based on a survey by Grigg et al. (5), anxiety and fear related to travel or to the carrier used for travel has been reported as the most prevalent behavior problem by 67% of the cat owners. In another owner survey, Mariti et al. (6) showed that 59% of cats were reported to exhibit signs of distress during car travel and 66% during veterinary visits. Pre-visit anxiolytic medications can be prescribed to make transport and veterinary visits less stressful for feline patients. The goal of pharmacologic intervention is to reduce anxiety during transportation and to enable patient-friendly, low-stress, physical examination (7). Currently, no licensed anxiolytic drugs are available for cats in the EU or the USA. Recently, studies concerning extra-label use of trazodone (8) and gabapentin (9) to reduce transport- and veterinary examination-related anxiety in cats have been published.

Pregabalin is a structural analog of neurotransmitter gamma-aminobutyric acid (GABA). Its mechanism of action is similar to gabapentin, but it is more potent and has favorable pharmacokinetic properties (10). The mode of action of pregabalin differs from benzodiazepines and other anxiolytic agents. It binds to the alpha-2-delta subunit of the voltage-dependent calcium channel in the central nervous system and decreases the release of several neurotransmitters, including glutamate and monoaminergic neurotransmitters, which are implicated in the pathophysiology of anxiety (11). A dose-dependent anxiolytic effect of pregabalin has been demonstrated in rodent models (12–14), and in humans (15) but not yet described in cats.

The objective of this pilot study was to evaluate the dosage, efficacy, and clinical safety of pregabalin in cats that are challenging to place into a carrier and/or distressed when transported by car.

## Materials and Methods

This study was a randomized, blinded, placebo-controlled, single dose, crossover study. The study procedures were performed at home by the cat owners. The study protocol was approved by the competent regulatory authority in Finland (Finnish Medicines Agency Fimea). The study was conducted according to the study protocol and the principles of Good Clinical Practice as defined by the Veterinary International Conference on Harmonization (VICH) Guideline (GL) number 9, and informed consent was obtained in writing from the owners prior to enrolment. The welfare, treatment, and care of the study animals were ensured by veterinary supervision, and the owners were able to contact the investigating veterinarians at any time during the study.

### Animals

Client-owned cats were screened during a phone interview by the investigating veterinarians at Orion Pharma, using an owner-directed questionnaire (Supplementary Table 1). All cats admitted to the study were identified as healthy (American Society of Anesthesiologists status I or II) by a practicing veterinarian chosen by the owner. Cats aged at least 1 year and weighing a maximum of 8 kg were eligible for the study. Because the human formulation of oral pregabalin used here had a concentration of 20 mg/ml, cats heavier than this would have had to be given large volumes, which could have led to poor compliance. Cats had to have a history of either being challenging to place and keep in a carrier or fearful and/or anxious when transported by car. These behaviors were verified at the baseline. Cats were excluded from participating if they were being treated with other psychoactive medications, homeopathic remedies, pheromonal products, supplements, or a special diet to control anxiety. Other reasons for exclusion were pregnancy, lactation, concurrent participation to any other clinical study, and any other condition or situation which could disturb the conduct of the study, for example, owner's inability to administer the study products or conduct the car transportations.

### Treatments

**Figure 1:**
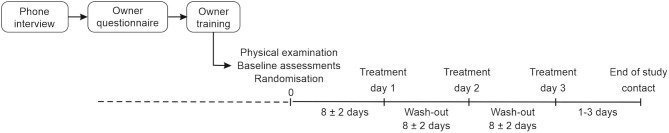
Study design of the crossover pilot clinical study. Pregabalin at a dose of 5 and 10 mg/kg and placebo were given in a randomized order to cats on three treatment days.

At baseline, behavioral assessments and a health check were performed. Cats were also given tap water orally using a syringe to mimic the study procedure. If eligible, a cat was enrolled and treated on three separate days, with wash-out periods of 8 ± 2 days between each treatment day (Figure 1). The cats received pregabalin oral solution (Lyrica, Pfizer), at a dosage of 5 and 10 mg/kg, and placebo, matched to the active treatment regarding taste, odor, and appearance. The treatments were administered in a randomized order using a three-period, three-treatment Williams crossover design. Randomization was conducted by an independent randomization specialist before the study start using computer software. The dosages were selected based on a previous non-clinical study in laboratory cats conducted by Orion Pharma. The study treatments were administered directly into to the cat's mouth with a syringe at home by the owner, who was blinded to the study treatments. The treatments were administered without food, but a small treat could be given to the cat after dosing. The owner was trained by the investigators to administer the study product and received written dosing instructions and a diary to report the study treatment administrations, assessments, and observations. An end-of-study contact followed 1 to 3 days after the last treatment day.

### Assessments

At baseline and during the three treatment days, 90 ± 15 min after study treatment administration, the cats were placed into a carrier and transported in a car for 20 min. Treatment administration, placing the cat into the carrier, transportation, opening the carrier after return, and a short time at 60 min after return were video recorded by the owner. Individuals making efficacy assessments, that is, owners and an external observer, were blinded to the study treatment.

The owner assessed the efficacy during the treatment days compared to baseline on the following variables: the overall effect of the study treatment during each treatment day, the ability to perform the procedures (placing the cat into the carrier at home and transportation of the cat in a car), and signs of stress, anxiety, and/or fear when placing the cat into a carrier and during transport. Additionally, the owners assessed the cat's activity and the usability of the product. Adverse events were recorded by the investigators based on owner interview and video observations.

Categorical scales were used in the assessments. Treatment effect was scored as “excellent,” “good,” “some effect,” “no effect,” or “worse.” The ability to place the cat into the carrier was scored as “excellent,” “good,” “fair,” “poor,” “very poor,” or “not possible.” For transportation in a car, a scale of “excellent,” “good,” “fair,” “poor,” and “very poor” was used.

The signs of distress, anxiety, and/or fear included vocalization, abnormal activity/restlessness/pacing, resistance/destructive behavior, escaping/evading/hiding, inappropriate urination, inappropriate defecation, panting/intense breathing, vomiting, licking/self-grooming, freezing/decreased motor activity, salivation, and sweaty paws. The severity of these signs was rated each as “absent,” “mild,” “moderate,” or “severe” (Table 1). The sum of the signs was calculated over all signs using their numerical severity rating.

**Table 1:** Rating of the signs of distress, anxiety, and/or fear by the cat owner.

**Numerical rating**	**Severity**
0	Absent
1	Mild
2	Mild
3	Moderate
4	Moderate
5	Severe
6	Severe

To assess the potential sedative effect of the treatment, the cat's activity was scored as “very calm/sleeping,” “calm,” “neutral,” “active,” or “very active.” The usability of the product was assessed using a scale of “very easy,” “easy,” “somewhat difficult,” and “very difficult.”

An external, independent, trained observer blinded to the owner assessment and the study treatment assessed the overall efficacy during each treatment day compared to baseline based on video recordings. The external observer scored the effect of the study treatment using the same rating scale as the owner. In addition, signs of anxiety and fear were assessed by the external observer either by frequency or duration depending on their nature according to an ethogram she created suitable for video assessment of the cats (Table 2).

**Table 2:** The ethogram used by the external observer for video assessment of cats.

**Signs assessed by duration (seconds)**	**Description**
Active interaction	Any behavior performed when interacting with the owner including active physical contact, sniffing, rubbing, close visual inspection, and gentle oral examination such as licking
Avoiding pet carrier	Get stuck, try to escape/break free while the owner tries to put the cat into the pet carrier (active resistance, but not aggressive—not biting, no scratching, no hissing)
Crouched position	Crouching. A pronounced lowering of the posture
Ears flattened	Ears flattened and back
Eating	Eating food
Exploration	Motor activity directed toward physical aspects of the environment, including sniffing, and gentle oral examination such as licking
Eyes closed	Sitting, standing, or lying down (the head does not rest on the ground) with eyes closed
Grooming	Action of cleaning the body surface by licking, nibbling, picking, rubbing, scratching, etc., directed toward the animal's body (self-grooming)
Hiding	Hiding
Locomotion	Walking around without exploring the environment
Not visible	Not visible (during these periods, activities like vocalizing, scratching, and chewing were identified and recorded by the sound of the activity)
Oriented to the environment	Sitting, standing, or lying down (the head does not rest on the ground) with obvious orientation toward the physical or social environment, including sniffing, close visual inspection, distant visual inspection (vigilance or scanning)
Passive behavior	Lying down with the head on ground without any obvious orientation toward the physical or social environment
Passive interaction	Sitting, standing or lying down during owner interaction or manipulation
Play	Any vigorous or galloping gaited behavior directed toward a toy; including chewing, biting, shaking from side to side, scratching or batting with the paw, chasing rolling balls, and tossing using the mouth. Although the cat may take the objects into its mouth, destruction is not included in this category
Pupils dilated	Mydriasis
Purring	Purring
Panting	Increased frequency of inhalation and exhalation often in combination with opening of mouth
Scratching	All active behaviors resulting in physical contact with the cage/door, including scratching the cage/door with the paws, jumping on the cage/door, handling with the forelimbs
Salivating	Salivation
Tail close to body	Lowered position of tail close to the body
Vocalization	Any form of vocalization, including: meowing, moaning, mewing, etc.
Withdraw	Avoiding interaction with the owner by running, moving away, very clearly turning away, or looking away
Elimination	Defecation or urination in sitting or standing position
Lip licking	Part of tongue is shown and moved along the upper lip
Shake off	Shaking the body to release stress
Swallowing	Swallowing
Vomiting	Vomiting
Yawning	Yawning

### Statistics

Due to the exploratory nature of this pilot study, neither a formal statistical hypothesis was defined nor a formal sample size calculation was performed. All cats entered into the study that received at least one dose of the study treatment were included in the analyses. Commercially available software SAS for Windows version 9.4 (SAS Institute Inc., Cary, NC, USA) was used.

All efficacy variables were analyzed in terms of absolute and/or change from baseline, and/or sum scores. Pregabalin was compared to placebo using a generalized linear model for ordinal data, with a cumulative logit as a link function, in which treatment and day were used as fixed effects. The correlation within cats was modeled as a repeated effect. Analyses were adjusted for a carry-over effect when a significant carry-over effect was found. The results were reported as odds ratio (OR) with 95% confidence interval (CI). The correlation between owners' and external observer's assessments of the overall effect of the study treatment was calculated using Spearman's rank-order correlation coefficient (r_s_). The change from baseline in individual signs and the sum of signs of distress, anxiety, and/or fear assessed by the owner was reported with mean (95% CI) and analyzed with a linear mixed model. The reliability of the external observer was assessed by a board-certified behaviorist using randomly selected 20% of the recordings using r_s_. The external observer's assessment of the duration or frequency of each sign was separately analyzed with a linear mixed model. Baseline data were used as a covariate, when available. Differences were considered to be statistically significant with *p* < 0.05.

## Results

No significant differences were found between pregabalin 5 and 10 mg/kg groups in efficacy analysis (Supplementary Table 2). Therefore, the efficacy results are reported as pregabalin groups combined.

### Animals

Fifteen cats were screened for the study. Two cats were withdrawn before administration of the first study treatment, leaving 13 cats that were enrolled. Seven cats were female and six were male. Their median (range) age was 3.3 years (1.0 to 8.9 years), and weight was 4.2 kg (4 to 7 kg). All cats, except one female, were neutered. Two cats discontinued the study after the first treatment day: one because the cat could not be placed into the carrier, and the other which had access to outdoors. Thus, 11 cats received all the study medications and the 2 cats received only one dose of pregabalin. The mean (min, max) actual dose was 5.1 mg/kg (4.8 mg/kg, 5.3 mg/kg) and 10.0 mg/kg (8.1 mg/kg, 10.7 mg/kg) for 5 and 10 mg/kg doses, respectively. In 29% (10/35) of the administrations the cat salivated or spilled out part of the dose. However, the overall treatment compliance was good as 71% (25/35) of the treatment administrations were successful.

### Owner Assessments

**Figure 2:**
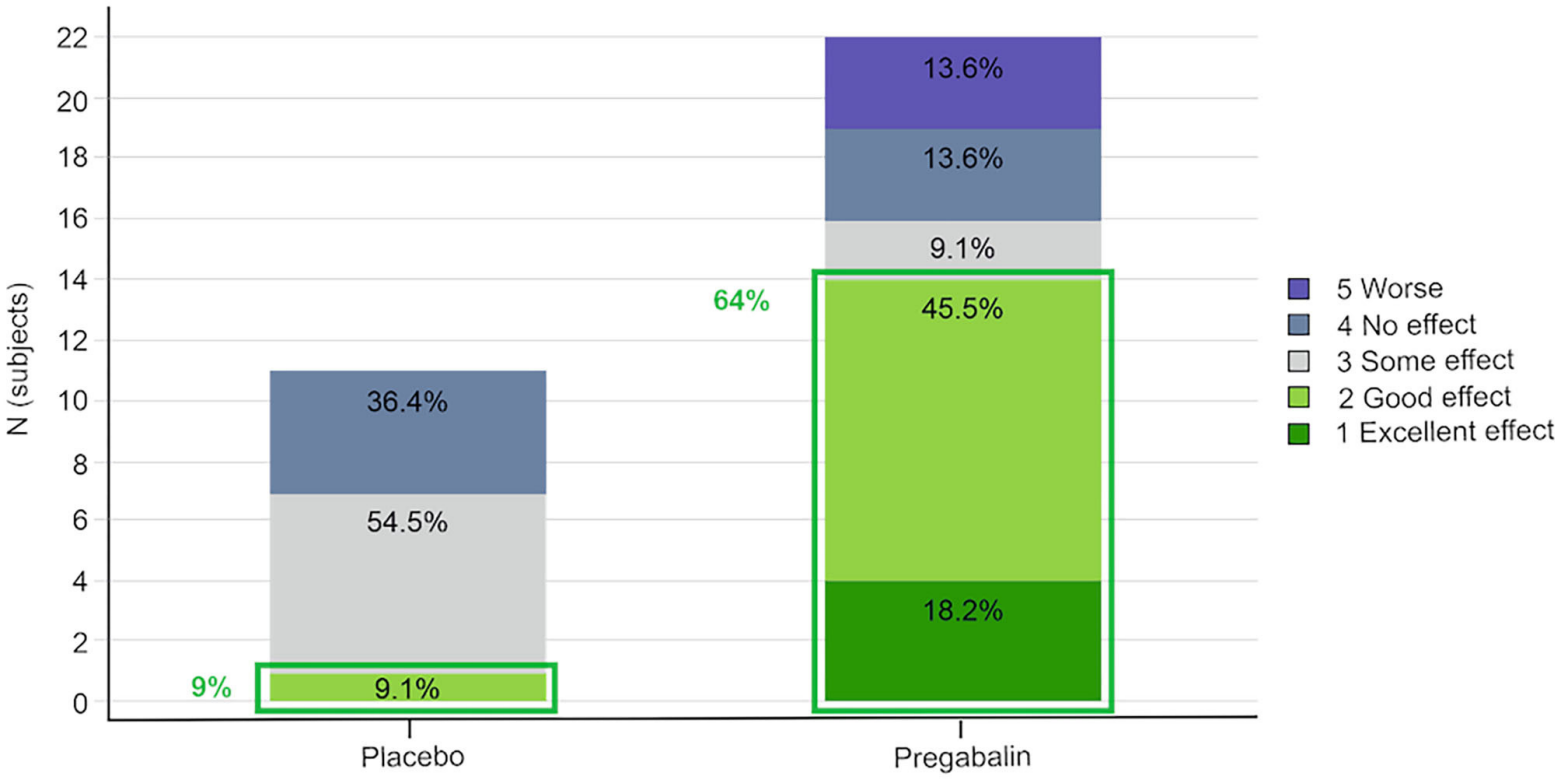
Owner's assessment of the effect of the study treatment. Pregabalin 5 and 10 mg/kg groups combined.

Overall, 64% (14/22 dosings) of the pregabalin treated cats (5 and 10 mg/kg groups combined) and 9% (1/11) of the placebo treated cats scored “excellent” or “good” for the overall effect of the study treatment (Figure 2).

The owners' assessment of the treatment effect (pregabalin groups combined vs. placebo) was significant [OR 4.2 (95% CI 1.1–16.6); *p* = 0.04]. However, a statistically significant carry-over effect was observed, as the cats randomized to receive pregabalin in the first or second period scored better results in the following period(s). When the model was adjusted for carry-over, the treatment effect was no longer significant [OR 2.4 (95% CI 0.6–8.9); *p* = 0.21].

In cats treated with pregabalin (groups combined), 83% (19/23) scored “excellent” or “good” in the ability to place the cat into the carrier compared to 73% (8/11) of the placebo treated cats. There was no statistically significant treatment effect [OR 1.3 (95% CI 0.6–3.0); *p* = 0.48].

In the ability to transport the cat in a car, 59% (13/22 dosings) of the pregabalin treated cats (groups combined) and 27% (3/11) of the placebo treated cats scored “excellent” or “good.” The treatment effect was not statistically significant [OR 2.8 (95% CI 1.0–8.3); *p* = 0.06].

**Figure 3:**
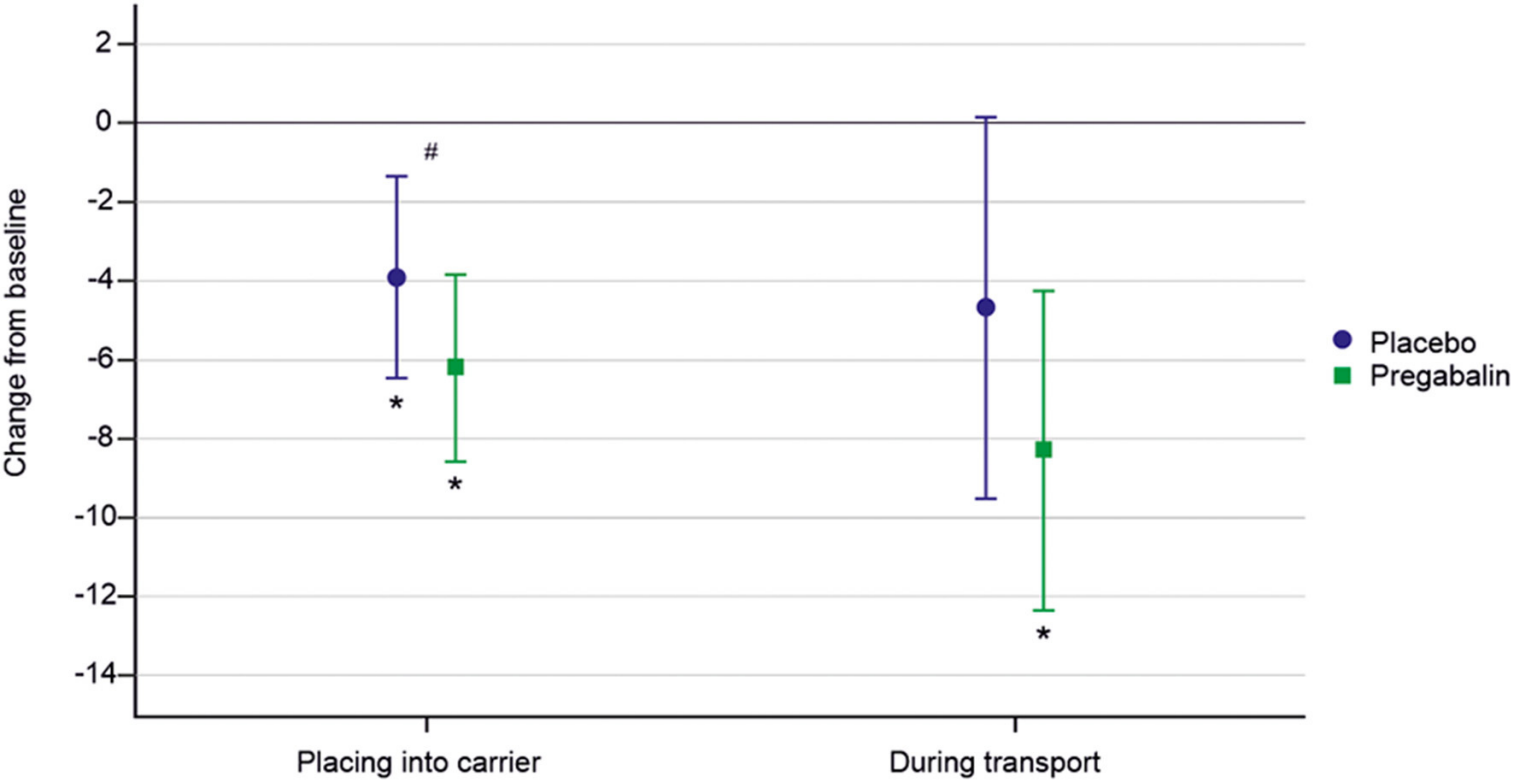
Model-based estimates of mean (95% CI) change from baseline in owner's assessment of signs of distress, anxiety, and/or fear. Pregabalin 5 and 10 mg/kg groups combined. *Indicates a significantly different value compared to baseline. ^#^Indicates a significant difference between treatments.

Figure 3 presents model-based estimates of mean (95% CI) change from baseline of sum of owner's assessment of signs of distress, anxiety, and/or fear. A significant (*p* < 0.01) decrease in sum of owner-rated signs of distress from baseline was seen when placing the cat into the carrier for both pregabalin and placebo, but this effect was seen only for pregabalin during transportation. A significantly larger decrease from the baseline in the sum score was seen for pregabalin compared to placebo for placing the cat into the carrier with mean decrease of −2.5 [(95% CI −4.3 to −0.7); *p* = 0.01]. This effect was not seen during transportation [−3.8 (95% CI −8.7 to 1.1); *p* = 0.12].

In the evaluation of individual signs of anxiety, fear, and stress during transportation, there was significantly less vocalization [mean −1.3 (95% CI −1.9 to −0.7); *p* < 0.01], abnormal activity/restlessness/pacing [mean −0.7 (95% CI −1.2 to −0.2); *p* = 0.01], and panting/intense breathing [mean −0.9 (95% CI −1.7 to −0.1); *p* = 0.03] after pregabalin treatment compared to placebo. There was significantly less vocalization after pregabalin treatment when placing the cat into the carrier [mean −0.6 (−1.0 to −0.2); *p* < 0.01].

After receiving pregabalin, cats were statistically significantly less active compared to placebo when placed into the carrier [OR 4.1 (95% CI 1.0–16.2); *p* < 0.05]; however, this effect was not found during transportation [OR 2.8 (95% CI 0.5–16.3); *p* = 0.24].

The owners assessed the administration of the product to be “difficult” or “very difficult” in 74% (25/34) of all administrations, while 26% (9/34) reported it to be “easy” or “very easy.” The volume of the administered product varied from 0.5 to 3.5 ml based on the dose and cat's weight. The difficulties in administration were not related to the dosing volume.

### External Observer Assessments

There was a significant correlation between the owners' and the external observer's assessment on the effect of the study treatment (r_s_ = 0.63, *p* < 0.01). The owners tended to assess the study treatment to be effective more often than did the external observer. Based on the video assessment, cats showed significantly less vocalization [mean −111.0 (95% CI −169.8 to −52.1); *p* < 0.01] and swallowing [mean −5.8 (95% CI −8.7 to −3.0); *p* < 0.01] and significantly more hiding [mean 0.2 (95% CI 0.1–0.3); *p* < 0.01] and passive interaction [mean 24.7 (95% CI 9.3–40.1); *p* < 0.01] when given pregabalin compared to placebo.

The inter-observer reliability was statistically significant for 17 of the signs in which r_s_ ranged from 0.65 to 0.94 (*p* < 0.05) and insignificant for 4 of the signs with r_s_ 0.10–0.59. For the remaining signs, sparse data precluded meaningful analysis.

### Safety

No serious adverse events were reported. The most common adverse event was transient ataxia, which was assessed as mild in 7 (58%) and 5 (42%) cats and as moderate in 1 (8%) and 7 (58%) cats with the doses 5 and 10 mg/kg, respectively. Muscle tremor and anxiety were reported once each (8%) with 10 mg/kg. There was no connection with the actual dose (mg/kg) and severity of ataxia. All adverse events had resolved by the next day when the investigators contacted the owners. No adverse events were reported with placebo.

## Discussion

Despite the recommendation by the veterinary associations that every pet should have at least one visit annually for preventive care (4, 16), ~45% of cat owners do not take their cat to a veterinarian (17), most likely due to difficulty in transporting the cat (3, 4). As a significant decrease in some signs of anxiety and fear was observed, this pilot study suggests that a single dose of pregabalin given by the owner at home could make the transportation easier for the cat and the owner and thus enable regular veterinary visits. However, as this was a small study, the results warrant a clinical study in a larger population of anxious cats.

Vocalization decreased substantially after pregabalin treatment compared to placebo during transportation and was likely the easiest for the owners to assess. Restlessness and panting were also significantly reduced. According to the external observer's assessment, vocalization and swallowing decreased the most when receiving pregabalin. These findings are in agreement with the signs of fear and anxiety reported for cats also in other studies (18–20).

As the external observer assessed the cat's behavior using a predefined ethogram, somewhat different signs were assessed compared to the owner. However, both the owners and external observer noted the same improvement in vocalization, which was the most prevalent sign observed. Hiding was only observed in a few cats, so the small increase in hiding (less than a second) in cats receiving pregabalin, although statistically significant, is likely not clinically or biologically relevant.

Owners assessed the treatment effect for the ability to place the cat into the carrier separate from that of the car transportation. When comparing pregabalin to placebo, a hint of positive treatment effect could be seen for transportation, which was supported by significant decrease in some signs of anxiety and fear after pregabalin treatment. In this population, based on the baseline data, it is clear that the main problem was transportation and not placement into the carrier. The demanding study design included several car rides for the cat with 1-week intervals, so owners who struggled with placing cats into carriers appear to have elected not to participate. A parallel design study with only one treatment period per cat would likely relieve this impediment. In general, a high number of trips has been reported to decrease owner willingness to participate in clinical studies (21).

Generally, owner observations of their pet's behavior, when they are instructed about the behaviors to observe, can be regarded as reliable (22–25). This pattern was shown in this pilot study. There was good correlation between owners' and the external observer's assessments of the overall treatment effect. Given the objectivity of the blinded external observer's assessment when evaluating the videos and the positive inter-observer reliability, owner assessment was deemed reliable and valid.

The two doses of pregabalin did not differ from each other significantly regarding efficacy; however, in safety assessments some differences were observed. Overall, pregabalin was well-tolerated, and the 5 mg/kg dose demonstrated a superior safety profile to the higher dose in the number and severity of adverse events.

The most frequently reported adverse event was transient ataxia, which is also reported with gabapentin (9). Esteban et al. (26) have studied pharmacokinetics of a single dose of pregabalin in oral capsules in healthy cats. In that study a dose of 4 mg/kg produced plasma concentrations in the cats that were reported to be similar to those considered efficacious for control of seizures in human patients with epilepsy. However, 4 of the 6 cats in that study showed signs of moderate sedation (26). Sedation is a known side effect in human patients treated with pregabalin (27). Signs related to the potential sedative effect, like ataxia and decreased activity, were observed also in our study. However, the signs associated with any sedative effect in our clinical study in client-owned cats were mostly mild, which may be due to the presence of travel-related anxiety. In the study by Esteban et al. (26) the cats were non-anxious, healthy, laboratory animals accustomed to handling and their familiar surroundings, which may be the reason for more severe signs of sedation at the lower dose reported in the study.

The usability of the formulation licensed for humans used in this study was poor, as most of the owners assessed it to be difficult or very difficult to administer. The administered volume did not seem to constitute the main difficulty in administration, but the taste and/or odor of the commercial strawberry flavor used in the human formulation may not be pleasant for cats, who generally prefer fish, liver, meat, yeast, and sour/acidic flavors (28).

The major limitations of the study are the small sample size and the observed carry-over effect. Learning/habituation by the cat and the owner due to several treatment days with 1-week intervals probably led to the observed carry-over effect, which is seen as an inherent problem in crossover studies (29). When studying clinical behavior, a parallel study design and larger study population might be more suitable. In a crossover study the carry-over could have been diminished with longer wash-out periods and including more than one placebo treatment day randomly in the study.

### Conclusions

This clinical pilot study showed that pregabalin decreases some signs of anxiety and fear associated with car transportation in cats. Such effects may improve the welfare of cats and aid owners in bringing their cat to the veterinarian. The clinical safety of pregabalin in client-owned cats was good, but the user-friendliness of the commercial human formulation used in this study was poor. A feline-specific formulation and further clinical data are warranted.

## Data Availability Statement

The raw data supporting the conclusions of this article will be made available by the authors, without undue reservation.

## Ethics Statement

The study was conducted in Finland and the clinical trial application was approved by the Finnish Medicine Agency (Fimea). The Directive 2010/63/EU on the protection of animals used for scientific purpose does not apply to veterinary clinical trials required for the marketing authorization of a veterinary medicinal product. Separate Ethical Committee approval was therefore not needed. The study was conducted according to the principles of Good Clinical Practice as defined by the VICH GL 9. Informed written owner consent for the use of each animal was obtained prior to any study-specific procedures. The welfare, treatment and care of study animals were ensured by veterinary supervision.

## Author Contributions

TL, MK, and MS contributed to the conception, design, and conduct of the study. JA contributed to the design of the study and performed the statistical analysis. CP contributed to the design and conduct of the study. KO contributed to the conception and design of the study. TL wrote the first draft. MK, MS, JA, CP, and KO contributed to writing and editing. All authors contributed to manuscript revision, read, and approved the submitted version.

## Conflict of Interest

This study received funding from Orion Pharma, which is the employer of TL, MK, MS, and JA. The funder had the following involvement with the study: TL, MK, and MS: conception, design and conduct of the study, and writing. JA: design, statistical analysis and writing. CP (design, conduct of the study and writing) and KO (conception, design of the study and writing) are paid consultants of Orion Pharma.

## Publisher's Note

All claims expressed in this article are solely those of the authors and do not necessarily represent those of their affiliated organizations, or those of the publisher, the editors and the reviewers. Any product that may be evaluated in this article, or claim that may be made by its manufacturer, is not guaranteed or endorsed by the publisher.
