# Supplementary material for: Efficacy of a Single Dose of Pregabalin on Signs of Anxiety in Cats During Transportation—A Pilot Study
Source: Front Vet Sci. 2021 Sep 1;8:711816. doi: 10.3389/fvets.2021.711816 (PMC8440915; doi:10.3389/fvets.2021.711816)
Supplement: Supplementary file 1 [file Data_Sheet_1.PDF]

Table 1 Screening questionnaire for cat owners

<table>
<tr><td colspan="2">Owner's name</td><td colspan="2">Phone number
Work
Private</td><td colspan="2">Home address

Email</td></tr>
<tr><td colspan="2">Cat's name</td><td colspan="2">Breed</td><td colspan="2">Cat's colour / description</td></tr>
<tr><td colspan="2">Cat's birth date</td><td colspan="2">☐ Male
☐ Female</td><td colspan="2">Castrated / sterilized
☐ No
☐ Yes</td></tr>
<tr><td colspan="3">Pregnancy/lactation during the study (only females)
☐ No
☐ Yes</td><td colspan="3">Bodyweight (estimate):</td></tr>
<tr><td colspan="3">Is your cat accustomed to handling?

Do you think that dosing of oral solution into cat's mouth with a syringe can be managed?</td><td colspan="3">Does your cat live mainly
☐ outdoors (free access daily)
☐ indoors (does not go out on its own)
If living indoors, does he/she go outdoors in harness/leash/have an outdoor cage etc.?</td></tr>
<tr><td colspan="3">Illnesses and their duration:</td><td colspan="3">Medications and their duration:</td></tr>
<tr><td colspan="3" rowspan="2">Has the cat's anxiety and stress related to travelling been treated somehow earlier (medication/therapy)?

If yes, what has been the result?</td><td colspan="3">Does your cat defecate regularly?
☐ Yes
☐ No
How often on average?</td></tr>
<tr><td colspan="3">Do you have other cats in your household?</td></tr>
<tr><td colspan="6">Do the other cats in your household suffer from travel anxiety?
☐ No
☐ Yes (please, describe)
With how many cats you would like to/could participate to the study?</td></tr>
<tr><td colspan="6">If your cat is suitable for the study, please give the time points when you are able to participate to the owner information and training meeting. The meeting takes about 3-4 hours.</td></tr>
<tr><td colspan="6">The assessments of the cat are conducted at home once a week for about 4 weeks during the study. Please, inform if you are planning to have a vacation, travel or other obstacles that could interrupt the study conduct.</td></tr>
<tr><td colspan="6">Other comments:

Date:</td></tr>
</table>

The original owner-directed screening questionnairy was in Finnish, which is the native language of the responders.

Owner's assessment of cat's signs

Assess your cat's signs of distress, fear and anxiety when placing the cat into a carrier.

Please, assess the signs/behavior of your cat **when placing the cat into a carrier** (without any treatment) by marking (x) all suitable points in the table below.

**Signs/behavior**	Absent	Mild		Moderate		Severe	
	0	1	2	3	4	5	6
**Vocalization** (hissing, growling, crying, whining, snarling)							
**Abnormal activity** (frequent change of place, running around, restlessness /pacing)							
**Resistance** (struggling, scratching, biting, swatting)							
**Escaping, evading, hiding**							
**Inappropriate urination**							
**Inappropriate defaecation**							
**Panting, intense breathing** (e.g. tongue out)							
**Continuous licking/self- grooming**							
**Freezing/decreased motor activity**							
**Salivation**							
**Sweating paws**							
**Other**, specify behaviours							

Assess your cat's signs of distress, fear and anxiety during transportation

Please, assess the signs/behavior of your cat **during transportation** (without any treatment) by marking (x) all suitable points in the table below.

**Signs/behavior**	Absent	Mild		Moderate		Severe	
	0	1	2	3	4	5	6
**Vocalization** (hissing, growling, crying, whining, snarling)							
**Abnormal activity** (frequent change of place, running around, restlessness /pacing)							
**Destructive behaviour** (destroying/biting carrier or contents)							
**Escaping, evading, hiding**							
**Inappropriate urination**							
**Inappropriate defaecation**							
**Panting, intense breathing** (e.g. tongue out)							
**Continuous licking/self- grooming**							
**Freezing/decreased motor activity**							
**Salivation**							
**Sweating paws**							
**Other**, specify behaviours							

Please, choose the most suitable option

1. What situations cause the signs assessed in the tables above?

- ☐ Placing the cat into a carrier
- ☐ Car transportation
- ☐ Other, please describe

____

2. Are there such car transportations that do not cause signs of distress, fear or anxiety?

- ☐ No
- ☐ Yes, please describe

____

3. How long car transportations does your cat usually have?

- ☐ 4-8 hours
- ☐ 1-4 hours
- ☐ less than 1 hour
- ☐ less than 30 minutes

4. How often does your cat travel in a car?

- ☐ Several times a week
- ☐ Once a week
- ☐ Once a month
- ☐ Sporadically few times a year
- ☐ I try to avoid transport of my cat in a car

5. Do you have another cat or dog in your household?

- ☐ No other cat or dog
- ☐ Yes, a dog/dogs
- ☐ Yes, a cat/cats
- ☐ Yes, a dog/dogs and a cat/cats

6. How do you feel when you have to transport your cat in a car?

1------------2------------3------------4------------5

I am totally calm — I am very nervous/tensed

7. What does your cat feel (based on your assessment) when you have to transport her/him in a car?

1------------2------------3------------4------------5

She/he is totally calm — She/he is very nervous/tensed
