# Supplementary material for: Efficacy of a Single Dose of Pregabalin on Signs of Anxiety in Cats During Transportation—A Pilot Study
Source: Front Vet Sci. 2021 Sep 1;8:711816. doi: 10.3389/fvets.2021.711816 (PMC8440915; doi:10.3389/fvets.2021.711816)
Supplement: Supplementary file 2 [file Data_Sheet_2.PDF]

Table 2. Statistical analysis results comparing pregabalin 5 and 10 mg/kg groups to placebo and each other

Generalized linear model for ordinal data. Results adjusted for carry-over effect shown where significant carry-over effect was observed.

Variable	Model	Effect	Odds ratio	95% Confidence Interval	P-value
Owners' assessment of the treatment effect	Unadjusted for carry-over	Pregabalin vs. Placebo	4.20	(1.06, 16.60)	0.0407
		5 mg/kg vs. Placebo	3.22	(0.71, 14.61)	0.1293
		10 mg/kg vs. Placebo	5.47	(1.01, 29.61)	0.0485
		5 mg/kg vs. 10 mg/kg	0.59	(0.11, 3.06)	0.5288
	Adjusted for carry-over (5 mg/kg carry-over effect p=0.0274, 10 mg/kg carry-over effect p=0.8198)	Pregabalin vs. Placebo	2.36	(0.62, 8.91)	0.2069
		5 mg/kg vs. Placebo	1.77	(0.31, 10.01)	0.5159
		10 mg/kg vs. Placebo	3.13	(0.87, 11.24)	0.0808
		5 mg/kg vs. 10 mg/kg	0.57	(0.13, 2.49)	0.4526
Ability to place the cat into the carrier	Unadjusted for carry-over	Pregabalin vs. Placebo	1.33	(0.60, 2.97)	0.4843
		5 mg/kg vs. Placebo	0.92	(0.36, 2.36)	0.8648
		10 mg/kg vs. Placebo	1.92	(0.72, 5.13)	0.1912
		5 mg/kg vs. 10 mg/kg	0.48	(0.17, 1.39)	0.1730
Ability to transport the cat in a car	Unadjusted for carry-over	Pregabalin vs. Placebo	2.82	(0.97, 8.25)	0.0580
		5 mg/kg vs. Placebo	2.89	(1.06, 7.90)	0.0387
		10 mg/kg vs. Placebo	2.76	(0.75, 10.19)	0.1283
		5 mg/kg vs. 10 mg/kg	1.05	(0.42, 2.61)	0.9210
Activity when placed into the carrier	Unadjusted for carry-over	Pregabalin vs. Placebo	4.06	(1.02, 16.20)	0.0474
		5 mg/kg vs. Placebo	0.39	(0.08, 1.79)	0.2240
		10 mg/kg vs. Placebo	42.53	(5.71, 316.89)	0.0003
		5 mg/kg vs. 10 mg/kg	0.01	(0.00, 0.09)	<.0001
Activity during transportation	Unadjusted for carry-over	Pregabalin vs. Placebo	5.31	(0.93, 30.24)	0.0598
		5 mg/kg vs. Placebo	2.23	(0.55, 9.00)	0.2584
		10 mg/kg vs. Placebo	12.63	(1.32, 121.18)	0.0279

		5 mg/kg vs. 10 mg/kg	0.18	(0.04, 0.73)	0.0167
	Adjusted for carry-over (5 mg/kg carry-over effect p=0.9791, 10 mg/kg carry-over effect p<0.0001)	Pregabalin vs. Placebo	2.84	(0.49, 16.34)	0.2431
		5 mg/kg vs. Placebo	1.26	(0.20, 8.09)	0.8093
		10 mg/kg vs. Placebo	6.40	(0.81, 50.52)	0.0782
		5 mg/kg vs. 10 mg/kg	0.20	(0.03, 1.18)	0.0747

Linear mixed model for change from baseline analyses of owner's assessment of signs of distress, anxiety and/or fear. All results are from models unadjusted for carry-over, as no significant carry-over effect was observed.

**Variable**	**Time point**	**Label**	**Estimate**	**95% Confidence Interval**	**Pr > |t|**
Sum of signs of distress, anxiety and/or fear	Placing into carrier	Pregabalin vs. Placebo	-2.48	(-4.30, -0.65)	0.0108
		5 mg/kg vs. Placebo	-1.65	(-3.71, 0.42)	0.1108
		10 mg/kg vs. Placebo	-3.30	(-5.47, -1.14)	0.0051
		5 mg/kg vs. 10 mg/kg	1.66	(-0.49, 3.81)	0.1226
		Pregabalin change from baseline	-6.49	(-8.89, -4.10)	<.0001
		Placebo change from baseline	-4.02	(-6.59, -1.44)	0.0044
	During transportation	Pregabalin vs. Placebo	-3.81	(-8.69, 1.07)	0.1182
		5 mg/kg vs. Placebo	-2.01	(-7.60, 3.58)	0.4591
		10 mg/kg vs. Placebo	-5.62	(-11.37, 0.13)	0.0550
		5 mg/kg vs. 10 mg/kg	3.61	(-2.16, 9.37)	0.2056
		Pregabalin change from baseline	-8.60	(-12.80, -4.40)	0.0006
		Placebo change from baseline	-4.79	(-9.72, 0.15)	0.0567
Vocalisation	Placing into carrier	Pregabalin vs. Placebo	-0.57	(-0.98, -0.17)	0.0084
		5 mg/kg vs. Placebo	-0.47	(-0.93, -0.014)	0.0441
		10 mg/kg vs. Placebo	-0.67	(-1.16, -0.19)	0.0090
		5 mg/kg vs. 10 mg/kg	0.20	(-0.28, 0.68)	0.3918
	During transportation	Pregabalin vs. Placebo	-1.32	(-1.92, -0.72)	0.0002

		5 mg/kg vs. Placebo	-1.01	(-1.69, -0.33)	0.0061
		10 mg/kg vs. Placebo	-1.63	(-2.34, -0.92)	0.0001
		5 mg/kg vs. 10 mg/kg	0.62	(-0.09, 1.33)	0.0834
Abnormal activity/restlessness/pacing	During transportation	Pregabalin vs. Placebo	-0.65	(-1.16, -0.15)	0.0135
		5 mg/kg vs. Placebo	-0.52	(-1.10, 0.05)	0.0697
		10 mg/kg vs. Placebo	-0.78	(-1.38, -0.19)	0.0127
		5 mg/kg vs. 10 mg/kg	0.26	(-0.34, 0.85)	0.3733
Panting/intense breathing	During transportation	Pregabalin vs. Placebo	-0.90	(-1.71, -0.10)	0.0299
		5 mg/kg vs. Placebo	-0.81	(-1.73, 0.11)	0.0822
		10 mg/kg vs. Placebo	-1.00	(-1.94, -0.053)	0.0395
		5 mg/kg vs. 10 mg/kg	0.19	(-0.76, 1.13)	0.6831

Linear mixed model for change from baseline analyses of external expert’s assessment of signs of distress, anxiety and/or fear based on video recordings. All results are from models unadjusted for carry-over, as no significant carry-over effect was observed.

**Variable**	**Sign of distress / Model**	**Label**	**Estimate**	**95% Confidence Interval**	**Pr > |t|**
Signs of distress, anxiety and/or fear	Vocalisation	Pregabalin vs. Placebo	-110.97	(-169.84, -52.11)	0.0010
		5 mg/kg vs. Placebo	-105.40	(-171.60, -39.19)	0.0038
		10 mg/kg vs. Placebo	-116.55	(-184.88, -48.22)	0.0024
		5 mg/kg vs. 10 mg/kg	11.15	(-54.13, 76.44)	0.7178
	Swallowing	Pregabalin vs. Placebo	-5.85	(-8.71, -2.99)	0.0013
		5 mg/kg vs. Placebo	-5.73	(-9.13, -2.34)	0.0042
		10 mg/kg vs. Placebo	-5.96	(-9.24, -2.68)	0.0026
		5 mg/kg vs. 10 mg/kg	0.23	(-3.20, 3.66)	0.8840
	Hiding	Pregabalin vs. Placebo	0.23	(0.14, 0.33)	0.0007
		5 mg/kg vs. Placebo	0.04	(-0.06, 0.15)	0.3775
		10 mg/kg vs. Placebo	0.43	(0.31, 0.54)	<.0001
		5 mg/kg vs. 10 mg/kg	-0.39	(-0.51, -0.26)	0.0003

	Passive interaction	Pregabalin vs. Placebo	24.72	(9.32, 40.11)	0.0051
		5 mg/kg vs. Placebo	29.46	(13.65, 45.26)	0.0022
		10 mg/kg vs. Placebo	19.97	(-0.06, 40.01)	0.0506
		5 mg/kg vs. 10 mg/kg	9.48	(-9.31, 28.28)	0.2857
